# Mitigating adolescent social anxiety symptoms: the effects of social support and social self-efficacy in findings from the Young-HUNT 3 study

**DOI:** 10.1007/s00787-020-01529-0

**Published:** 2020-04-16

**Authors:** Tore Aune, Else Marie Lysfjord Juul, Deborah C. Beidel, Hans M. Nordahl, Robert D. Dvorak

**Affiliations:** 1 Faculty of Nursing and Health Sciences, Nord University, Levanger, Norway; 2 Faculty of Nursing and Health Sciences, Nord University, Namsos, Norway; 3 UCF RESTORES, University of Central Florida, 4111 Pictor Lane, Orlando, FL 32816 USA; 4 Department of Mental Health, NTNU, Trondheim, Norway; 5 Division of Psychiatry, St.Olavs Hospital, Nidaros DPS, Trondheim, Norway; 6 Department of Psychology, College of Science, University of Central Florida, 4111 Pictor Lane, Orlando, FL 32816 USA

**Keywords:** Negative life events, Social support, Social self-efficacy, Social anxiety disorder, Social anxiety disorder symptoms, Intervention

## Abstract

Adolescents’ exposure to negative life events (NLEs) and potentially traumatic events is highly prevalent and increases their risk of developing psychological disorders considerably. NLE exposure has also been linked to the development of social anxiety disorder (SAD) among older children and young adolescents. Despite the relatively low treatment efficacy reported for children and adolescents suffering from SAD, few studies have addressed the extent to which resilience factors, such as social support and social self-efficacy, are associated with SAD symptoms. This study examined whether social support and social self-efficacy predict, and buffer against SAD symptoms using a large, population-based sample of adolescents, among whom a large proportion have experienced NLEs. The results reveal that NLEs are significantly associated with SAD symptoms, while social support and social self-efficacy are both negatively associated with SAD symptoms. Only the NLEs × social support interaction significantly predicted SAD symptoms, with social support attenuating the association between NLEs and SAD symptoms. Moreover, increases in both social self-efficacy and social support were associated with reduced SAD symptoms, over and above variance explained by social support alone. Our cumulative results suggest that interventions that can modify both social support and social self-efficacy may help reduce SAD symptoms in at-risk adolescents.

## Introduction

Trauma exposure is highly prevalent among both adults and adolescents [1, 2], with both groups showing exposure-related symptoms including withdrawal, sleeping difficulties [3], posttraumatic stress disorder (PTSD) [4], and depression and anxiety symptoms [5], often causing substantial impairment [6]. Several studies [7–9] have also shown that adolescents exposed to negative life events (NLEs), i.e., extraordinary experiences with the potential to alter one’s social life [10], or potentially traumatic events (PTEs), i.e., experiences which are life-threatening or pose a significant threat to a person’s physical or psychological wellbeing [11], at any point during their lives have a markedly increased risk of developing psychopathology.

Frazier et al. [1] reported that 85% of their U.S. sample of undergraduate students endorsed having experienced at least one NLE during their lifetime. Exploring a population-based sample of 11–14 year-old students, Aune and Stiles [12] showed that 73% had experienced at least one NLE during the past year, while 33% had experienced three or more NLEs during a one-year period. These figures indicate that experiencing NLEs and PTEs is common among older children, adolescents, and young adults [12–14]. Cumulative NLE exposure is a powerful predictor of general mental health problems [15], emphasizing the importance of examining the totality of stressful experiences rather than isolated adverse exposure. Aune and Stiles [12] have also shown that NLEs predict the development of social anxiety disorder (SAD), even among older children and young adolescents. Comorbidity between NLEs and SAD has been documented in older samples [16–19].

SAD is associated with significant educational, occupational, and social functioning impairment [20] and is one of the most prevalent chronic [21] and pervasive disorders [22] among adolescents [23]. Recognition of the clinical importance of SAD among children and adolescents [24] has fostered the development of clinical treatments, including Social Skills Training; Enhancing Social Competence in Children and Adolescents (SST) [25], Cognitive Behavior Group Therapy for Adolescents (CBGT-A) [26], Social Effectiveness Therapy for Children (SET-C) [27], the “Lynx-program,” [28] and school-based intervention programs, such as Skills for Academic and Social Success (SASS) [29] and the Norwegian Universal Prevention Program for Social Anxiety (NUPP-SA) [30]. Despite these promising treatment studies, treatment efficacy is generally only 40–50% [24, 31]. Though SAD treatment efficacy is underwhelming, and the disorder is often chronic; therefore, in addition to continuing to develop better interventions, research examining factors that may protect against the development of SAD symptoms and clinical-level SAD is warranted [24].

NLEs often result in negative outcomes. However, a relatively vast body of evidence demonstrates that trusted networks are vital if young people are to recover from the impact of adversity [32]. Luthar and colleagues [33] concluded that resilience, a phenomenon of positive adaptation among youth who are considered vulnerable for developing later psychopathology [34], is fundamentally based on relationships. Masten [35] showed that three out of ten key resilience factors are consistently related to interpersonal support. Rutter’s [36] literature review further identified supportive social connections as the fundamental feature of resilience; social support has also been identified as an essential feature for both preventing and recovering from anxiety disorders, including PTSD specifically and mental health issues generally [36]. The social buffering hypothesis [37] suggests that social support reduces strain and buffers against the adverse impacts of stress and NLEs, thereby contributing to well-being. In contrast, a study related to the bombing in Oklahoma City [38] identified self-efficacy as a unique predictor of resilience following PTEs, independent of social support. Self-efficacy is the belief that one is capable of influencing, organizing, and executing the course of actions required to manage prospective events in one’s life [39, 40]. Moreover, both Bandura [41] and Muris [42] stressed the importance of assessing social self-efficacy among young people, i.e., an individual’s confidence in her/his ability to engage in social interactional tasks necessary to initiate and maintain interpersonal relationships [43]. Thus, self-efficacy, involving both cognitive and affective processes, may affect an individual’s insight into, and elaboration about, NLEs, increasing their ability to manage and recover from such events [44]. Alternatively, viewing self-efficacy as an independent resilience and protective factor, Schwarzer and Knoll [45] suggested that social support is indirectly associated with outcomes from NLEs because it facilitates the direct relationship between self-efficacy and mental health outcomes [46]. This model suggests that social support may facilitate increases in self-efficacy, which then facilitate reductions in PTEs and, secondarily, disorders such as PTSD [46, 47]. In contrast, self-efficacy may also facilitate and maintain social support, which would reinforce social resources and result in reduced mental health symptoms. This model indicates that social support, in contrast to self-efficacy, is primarily responsible for the reduction in mental health symptoms. Finally, the association between social support and self-efficacy may be bidirectional, with these two resilience factors bolstering one other and thus predicting mental health [45, 46].

The cumulative evidence shows that SAD is a prevalent and pervasive disorder among older children and adolescents [23], that treatment efficacy is generally low [24, 31], and that the disorder often becomes chronic when developed at an early age [21]. Furthermore, Aune and Stiles [12] have reported that experiencing NLEs, even at a young age, predicts the development of SAD. There is compelling evidence that social support buffers the development of psychopathology in general [36, 48], though the extent to which social support is related to the development of SAD is unclear. Further, self-efficacy is a unique predictor of mental health outcomes following NLEs [39, 46, 49] and Adams and colleagues [46] have suggested that social support enables self-efficacy and that social support is indirectly related to mental health only through perceived self-efficacy. However, the extent to which self-efficacy protects against SAD symptoms among adolescents who have experienced traumatic life events has not been explored.

The purpose of this study was to examine the extent to which NLEs predict SAD symptoms, via social support and social self-efficacy, individually and in conjunction. Based on previous research, we expected that NLEs would predict greater SAD symptoms. In contrast, both social self-efficacy and social support were expected to be associated with fewer SAD symptoms in those experiencing NLEs. Finally, we hypothesized that both social self-efficacy and social support would mitigate the association between NLEs and SAD symptoms.

## Methods

### Participants

The Young-HUNT 3 Survey is a cross-sectional HUNT 3 survey conducted in Norway’s Nord-Trøndelag county for which Holmen et al. [50] assessed 8677 (83% response rate) adolescents aged 13–19 years. Nord-Trøndelag county has a stable population of approximately 132,000 inhabitants living across 23 municipalities and serves as a representative sample of Norway with regard to geography, industry, income source and level, age distribution, morbidity, and mortality [51]. Schools in Norway are all integrated, which means that all children and adolescents attend the same schools, including students with learning, physical, and behavioral disabilities. For a more detailed description of the Cohort Profile of the Young-HUNT 3 Study, see Holmen et al. [50] and Skrove et al. [52].

### Procedures

Participating schools were the primary study sites for the Young-HUNT 3 survey. In Norway, all adolescents are expected to attend junior high school (ages 13–16 years) and then high school (ages 16–19 years). The principals of all 66 schools in the county gave their written consent for their school to participate. All students attending these schools, and their parents, were invited to participate in the study. The invitation included information about the study and the intended use of the collected data. A list of those who had dropped out of school or transitioned to an apprentice program was provided by the county school administration so that these individuals would also be invited to participate. Statistics from the Norwegian National Population Register were used to verify study data (e.g., birthdates, names, addresses, national identity numbers). Thus, the entire cohort of those aged 13–19 years who were living in the county were invited to participate.

Data collection for the larger study included self-report questionnaires, structured interviews, clinical measurements, and buccal smears; for the present analyses we present results from self-report questionnaires. Students completed the questionnaires during the school day. Each questionnaire was printed with a unique barcode (i.e., no names or other identifiers were included on the form) and sealed in a blank envelope by the student after completion.

### Measures

#### Trauma Index: Impact of Event Scale

A modified version of the Impact of Event Scale was used [53]. This scale consists of 11 individual items related to the question: Have any of the following happened to you? *(Someone in your family has been seriously ill; Death of a loved one; Catastrophe (e.g., fire, avalanche, tidal wave, hurricane, *etc*.); Serious accident (e.g., very serious car accident); Violently hurt (beaten or injured); Seen others violently hurt; Been put in sexually uncomfortable/abusive situations by someone about your age; Been put in sexually uncomfortable/abusive situations by an adult; Been threatened or physically harassed by other students at school over a long period; Received painful or frightening treatment while at the hospital due to an illness or injury; Experienced something else that was very frightening, dangerous or violent).* Each item was rated on a 3-point Likert scale for which only one option could be endorsed (1 = no; 2 = yes, last year; 3 = yes, in my lifetime). This scale was later re-coded so that 0 = no; 1 = yes, last year; 1 = yes, in my lifetime. A summed score of these 11 items was created (range 0–11), with higher scores indicating experiencing more traumatic events.

#### Social support and social self-efficacy

Social support and social self-efficacy were assessed using eight items selected from two factors included in the original Resilience Scale for Adolescents (READ). The READ includes 28-items, each rated on a 5-point Likert scale, with positively formulated items organized into five subscales: personal competence, social competence, social support, family cohesion, and structure [54]. Among a relatively large population-based sample (*N* = 6723) of 18–20 year-olds, von Soest, Mossige, Stefansen, and Hjemdal [55] found a good fit with this five-factor solution. Examining READ’s psychometric properties, Askeland and Reedtz concluded that the READ shows adequate psychometric properties and validity when correlated with measures of mental difficulties [56].

Bandura [41] and Muris [42] both indicated that assessing social self-efficacy among young people is important, as it relates to their ability to deal with social challenges. The social competence factor in the READ includes items such as: “*I easily make others feel comfortable around me*” or “*I easily find new friends*.” Further, the concept of social support among children and young adolescents includes positive involvement and support from family and friends. The family cohesion factor in the READ includes items such as: “*In my family we share views of what is important in life*” or “*I feel comfortable with my family.*”

In a study by Huang and Mossige [57], the family cohesion factor was strongly and positively associated with “parental care” and “close friendship,” and negatively associated with “parental overprotection.” Both family cohesion and social competence factors were significant and negatively associated (*p* < 0.01) with “symptoms of anxiety,” “depressive symptoms,” “suicidal ideation,” and “self-harm” [57]. In the same study, the correlation between READ-based social competence and family cohesion factors was *r* = 0.46 and both factors showed adequate Cronbach’s alpha (0.77 and 0.89 for social competence and family cohesion, respectively) [57]. In this study, these factors were combined and renamed Social Support (family cohesion) and Social Self-Efficacy (social competence). Exploratory factor analysis revealed two distinct factors and Cronbach’s alphas of 0.86 for Social Support and 0.82 for Social Self-Efficacy, indicating adequate internal consistency.

#### Social anxiety disorder symptoms index

The questionnaire included six items assessing SAD symptoms, each using a five-point Likert scale. Using an item analysis approach [58], these six items were selected from the Social Phobia and Anxiety Inventory for Children (SPAI-C) [59, 60] and the Social Phobia and Anxiety Inventory (SPAI) [61]; from these, a summed score was calculated. Cronbach’s alpha for this score was 0.84, indicating adequate internal consistency. Using the Anxiety Disorders Interview Schedule for Children (ADIS-C) clinical interview [62], the SAD symptom index significantly differentiated between those diagnosed within the full spectrum of SAD, sub-clinical SAD, and the performance-only specifier SAD [63].

The following items comprise the SAD symptoms index: *I feel anxious and don’t know what to do in an embarrassing situation; I feel anxious when I am with others and have to do something while they watch me do it (e.g., be in a play, play music, sports); I feel anxious when I have to speak or read aloud in front of a group of people; Before I go someplace where I’m going to be with people (e.g., a party, school, football game) I sweat, my heart beats fast, and/or I get a headache or stomachache; Before I go to a party or someplace with other people I think about what could go wrong (e.g., that I will make mistakes, seem dumb, and/or they will see how frightened I am); I feel anxious and don’t know what to do when I’m in a new situation.*

### Statistical analysis

The dataset for these analyses included *N* = 8216 participants. However, data for the measures analyzed herein were completely missing for *n* = 321 participants (3.9% of the total sample). In addition, data were missing within individual surveys, ranging from 0.1 to 1.1%. Participants with all data missing were excluded from analyses. For participants with some data, within-measures missing data were replaced using multiple imputations [64]. Multiple imputations followed a three-step process whereby a series of 10 datasets were created with missing values replaced using an Ordinary Least Squares approach. These mean values were then pooled across the imputed datasets. Finally, a series of regression models were constructed to provide parameter estimates across all 10 datasets with pooled estimates. Previous research shows that multiple imputations is more effective than other forms of data replacement (e.g., omission of missing values, mean substitution) and that it provides stable and unbiased parameter estimates [65]. STATA version 15 was used for all analyses.

Hypotheses were tested using a moderated regression analysis. All variables were grand mean centered prior to analysis. First, we controlled for the association between SAD symptoms and demographic variables (age, gender). Next, we examined the direct effects of social support, social self-efficacy, and NLEs on SAD symptoms. Then, we tested the extent to which social self-efficacy and social support moderated the association between NLEs and SAD symptoms. Finally, we examined the moderated effects of NLEs by calculating the simple slopes of SAD symptoms for NLEs at the high and low moderator variable levels [66]. Given the large sample size, we probed significant interactions at ± 2 standard deviation (SD) of the moderator. This approach allows for the calculation of simple slopes at high and low levels of the protective factors (social support and self-efficacy). Thus, the moderation analysis looks at the relationship between the predictor variables of interest (NLE) when individuals have very high and/or very low protective factors, which allows the examination of how protective factors buffer the association between NLEs and SAD symptoms.

## Results

All students (10,464) aged 13–19 years in Norway’s Nord-Trøndelag county were invited to participate in this population-based study; 5084 (48.6%) were women and 5380 (51.4%) were men. Altogether, 8216 participants filled in questionnaires. Due to missing data, a final *N* = 7895 (96.1% of the original sample) participants were included in analyses. Men (*n* = 199; 5.13%) were more likely than women (*n* = 122; 2.95%) to be excluded due to missing data (*χ*^2^ [1]  = 20.29, *p* < 0.001). Missing data were also associated with lower age (missing: *M* = 15.63, SD ± 1.78; not missing: *M* = 15.90, ± 1.74; *t*(2.68), *p* = 0.007, Cohen’s *d* = 0.15). Due to missing all other variables of interest, we were unable to examine differences in social anxiety, social support, self-efficacy, and NLEs. However, the observed differences were small, and only statistically significant due to the large sample size.

The mean ages for women (*n* = 4013) and men (*n* = 3882) were (*M* = 15.92, SD ± 1.76) and (*M* = 15.90, SD ± 1.74), respectively. There was no statistically significant difference in age (*p* = 0.18) or NLEs (*p* = 0.08) based on sex. Though men endorsed higher levels of social support (*t* [7893] = 6.47, *p* < 0.001, Cohen’s *d* = 0.26) and social self-efficacy (*t* [7893]  = 10.34, *p* < 0.001, Cohen’s *d* = 0.23), the magnitude of these effects were small. Overall, NLEs were not significantly correlated with social self-efficacy (*r* =  − 0.02, *p* = 0.053), but were significantly correlated with social support (*r* =  − 0.18, *p* < 0.001). There was a robust, positive correlation between social support and social self-efficacy (*r* = 0.48, *p* < 0.001). SAD symptoms showed significant positive bivariate correlations with social self-efficacy (*r* = 0.45, *p* < 0.001), social support (*r* = 0.31, *p* < 0.001), and NLEs (*r* = 0.19, *p* < 0.001). Table 1 shows the mean and SD values for female and male participants on each measure.

**Table 1:** Means and standard deviations (SD) of negative life events, social support, social self-efficacy, and social anxiety disorder symptoms experienced by female and male students

Sex	Negative life events	Social support	Social self-efficacy	SAD symptoms
	Mean (SD)	Mean (SD)	Mean (SD)	Mean (SD)
Females	1.30 (1.40)	16.58 (3.51)	15.07 (3.28)	12.37 (4.37)
Males	1.21 (1.40)	17.05 (3.06)	15.82 (3.41)	10.45 (4.05)
Total	1.25 (1.43)	16.81 (3.31)	15.44 (3.24)	11.42 (4.32)

### Primary analyses

The variables were entered in a stepwise regression analysis. In step 1, we controlled for the association between SAD symptoms, age, and sex (*F* [2, 7892] = 248.99, *p* < 0.001, *R*^2^ = 0.06). Older age and women were associated with higher rates of SAD symptoms. In step 2, SAD symptoms were regressed onto NLEs, social self-efficacy, and social support (*F* [5, 7889] = 552.35, *p* < 0.001, *R*^2^ = 0.26). This resulted in a significant improvement in overall model fit (LR *χ*^2^ [3] = 1886.68, *p* < 0.001, ∆*R*^2^ = 0.20). In this step, NLEs were positively associated with SAD symptoms, while both social support and social self-efficacy were negatively associated with SAD symptoms. In step 3, the NLEs × social self-efficacy and NLEs × social support interactions were added to the model. Only the NLEs × social support interaction significantly predicted SAD symptoms. Thus, the NLEs × social self-efficacy interaction was removed and the model re-estimated. The final model with the single interaction (see Table 2) fit the data well (*F* [6, 7888] = 461.85, *p* < 0.001, *R*^2^ = 0.26). The addition of this single interaction improved fit over the model without its addition (LR *χ*^2^ [1]  = 7.19, *p* = 0.007).

**Table 2:** Moderated regression analysis examining negative life events, social support, and social self-efficacy as predictors of social anxiety disorder symptoms

Model components	*R*^2^	*df*	*F*	*β*	*P*
Step 1	0.06	2, 7892	248.99		< 0.001
Age				0.07	< 0.001
Sex				− 0.17	< 0.001
Step 2	0.26	5, 7889	552.35		< 0.001
Negative life events				0.09	< 0.001
Social support				− 0.10	< 0.001
Social self-efficacy				− 0.38	< 0.001
Step 3	0.26	6, 7888	461.85		< 0.001
Negative life events × social support				− 0.03	0.007

Final model accounted for 26% of the variance in social anxiety disorder symptoms. All model steps, as well as all parameters within each model were statistically significant at *p* ≤ 0.007. The interaction of negative life events × social self-efficacy was initially tested. This interaction was non-significant and thus removed from the final model

**Fig. 1:**
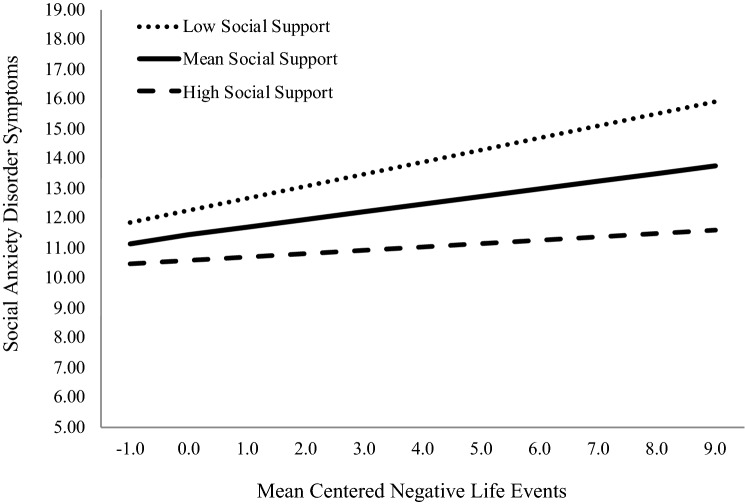
Simple slopes of social anxiety disorder symptoms on negative life events at high (+ 2SD) and low (− 2SD) levels of social support. Negative life events and social support were mean centered. At high social support, negative life events was not significantly associated with social anxiety disorder symptoms; however, at low social support there was a robust association between negative life events and social anxiety disorder symptoms

Next, we examined the buffering effect of social support on the relationship between SAD symptoms and NLEs by calculating the simple slopes of social support on NLEs at high (+ 2 SD) and low (− 2 SD) levels of social support (see Fig. 1). At high levels of social support, the association between NLEs and SAD symptoms diminished to the point where it was no longer statistically significant (*β* = 0.04, *p* = 0.08). In contrast, at low levels of social support the association between NLEs and SAD symptoms was potentiated (*β*  = 0.14, *p* < 0.001).

Social support appears to operate in two distinct ways. First, it has a robust inverse association with SAD symptoms (*β*  =  − 0.10, *p* < 0.001). Second, it attenuates the association between NLEs and SAD symptoms. Consequently, a change from mean levels of social support to high levels of social support results in reduced SAD symptoms, from a mean of 11.42–10.57 (Cohen’s *d* = 0.20). Similarly, increasing social self-efficacy by the same amount (2 SD) results in reduced SAD symptoms from a mean of 11.42–8.12 (Cohen’s *d* = 0.76). These findings suggest that interventions that can modify these variables could have a dramatic effect on SAD symptoms, potentially reducing adolescents’ mean symptom levels (as shown here, from 11.42 to 7.26 [Cohen’s *d* = 0.96]).

## Discussion

Overall, our results demonstrate that both social support and social self-efficacy are strongly associated with SAD symptoms among adolescents who have experienced NLEs. Specifically, social support seems to work in two distinct ways. First, it appears to be associated with lower SAD symptom levels generally. Second, when adolescents experience more frequent NLEs, it functions as a significant buffer, or protective mechanism, against SAD symptoms. In contrast, social self-efficacy was not significantly associated with aggregated levels of NLE’s in our study. However, social self-efficacy did emerge as a strong, general protective factor against SAD symptoms. Thus, while social self-efficacy has an additive protective effect on SAD symptoms, social support has a more synergistic protective effect, providing both direct protective effects against SAD symptoms as well as buffering the effects of adverse NLEs on SAD symptoms.

The cumulative picture emerging from these findings is that although social support is important, improving social self-efficacy among adolescents with SAD symptoms may also be an important intervention target. The general effect of manualized treatment for SAD in children and adolescents is relatively limited [24] because it focuses primarily on decreasing anxious arousal. However, given the limited social networks of most children and adolescents with SAD, decreasing arousal alone will not necessarily result in enhanced social relationships. Given the early onset of SAD, patterns of social avoidance also set in early, which hinder children in learning basic social interaction skills. Thus, a comprehensive behavioral treatment approach focusing on improving social skills as well as decreasing anxiety (i.e., the SET-C) has a treatment efficacy of 76% among older children and young adolescents [67], reinforcing the need for a multicomponent approach.

One possible interpretation of these results is that while social support is important, its impact is reduced if it does not improve social self-efficacy. Nevertheless, interventions that simultaneously potentiate social self-efficacy and social support appear to have an additive effect, as shown by the present study’s relatively high effect sizes. Longitudinal studies assessing both older children [21] and adolescents [18, 68] have shown a positive association between self-reported potentially traumatic social events and SAD. Thus, our results are consistent with those of previous studies that have demonstrated a link between NLEs and SAD symptoms. Our results are also partially consistent with those of Wong and Rapee [24], who developed a comprehensive integrated etiological and maintenance (IAM) model of SAD. This model proposes that multiple NLE experiences lead individuals to associate social evaluative stimuli with the greater threat, predicting higher SAD symptom levels. Thus, these authors have asserted that future research should investigate the extent to which multiple NLE experiences and social self-efficacy are related to social evaluative stimuli. Furthermore, Wong and Rapee’s [24] IAM model suggests that performance deficits, whether due to lack of age-appropriate social skills/knowledge or to anxiety/limited attention, serve as maintenance factors for SAD. The extent to which low social self-efficacy is associated with a lack of age-appropriate social skill, anxiety, and/or limited attention should also be further explored. Cumulatively, the data presented herein provide evidence that social self-efficacy is linearly associated with SAD symptoms and, thus, should be incorporated into the IAM model and further explored therapeutically.

This study has several strengths and limitations that must be addressed. We assessed a large population-based sample of adolescents, who participated at a high rate. The SAD symptom index is highly sensitive for distinguishing between adolescents with full-spectrum SAD and those with either subclinical SAD or SAD performance-only specifier [63]. However, the Impact of Events Scale consists of only 11 items; thus, it may have excluded some traumatic events. In addition, because we instructed adolescents to endorse only one of the three time-based response options, this scale cannot discriminate between those who experienced NLEs in past year versus more than one year ago. Finally, only social self-efficacy was assessed; a broader scale would have assessed self-efficacy more generally, while specific scales would have assessed academic or emotional self-efficacy. Nevertheless, both Bandura [41] and Muris [42] have stated the importance of assessing social self-efficacy as a predictor of mental health.

Additional studies will be needed to continue exploring the relations between social support and social self-efficacy, including how these protective factors are related to both the development and maintenance of SAD. We further encourage researchers to develop and examine treatment and prevention programs aimed at modifying adolescents’ experiences with social support and social self-efficacy. The established links between emotion, action, and self-efficacy perception [69] suggest that perceived self-efficacy may be a valuable target for SAD treatment. In this context, adolescents who believe they can master challenges will experience less anticipatory anxiety and thus participate more vigorously in social activities. Psychological interventions such as guided mastery therapy [69, 70] have been shown to enhance the perception of self-efficacy. For some adolescents, strengthening social support may not be an option; thus, empowering their perception of social self-efficacy may provide hope and confidence.

More effective treatments and prevention programs are needed for adolescents who suffer from SAD, which is one of the most prevalent, chronic, and pervasive mental health disorders, and the only mood or anxiety disorder that is consistently associated with premature drop-out from school [22]. Interventions empowering both social support and social self-efficacy appear to have the potential for significant impact.

### Data access

The HUNT study invited those aged 13–100 years to participate in three surveys between 1994 and 2008, with the latest survey (HUNT 4) administered beginning in 2017. Comprehensive data have been collected from more than 125,000 individuals who have participated at least once, and biological materials have been collected from 78,000 of these participants. Data are stored in the HUNT databank, and biological material are stored in the HUNT biobank. The HUNT Research Centre has permission from the Norwegian Data Inspectorate to store and handle these data. The key identifier in these databases is the personal identification number given to all Norwegians at birth or immigration; deidentified data are sent to researchers upon approval of a research protocol by the Regional Ethical Committee and HUNT Research Centre. To protect participants’ privacy, the HUNT Research Centre aims to limit data storage outside the HUNT databank and cannot deposit data in open repositories. The HUNT databank maintains detailed records regarding all data exported for various projects, which staff can produce upon request. With HUNT Research Centre application approval, there are no restrictions on data export. For more information, see: https://www.ntnu.edu/hunt/data.
